# P02 Antimicrobial susceptibility of microorganisms isolated in community-acquired pneumonia in drug-dependent patients

**DOI:** 10.1093/jacamr/dlad066.006

**Published:** 2023-06-14

**Authors:** Oksana Viltsaniuk, Yuriy Mostovoy, Oleksandr Viltsaniuk

**Affiliations:** National Pirogov Memorial Medical University, Vinnytsya, Ukraine; National Pirogov Memorial Medical University, Vinnytsya, Ukraine; National Pirogov Memorial Medical University, Vinnytsya, Ukraine

## Abstract

**Objectives:**

To investigate the nature and susceptibility of community-acquired pneumonia (CAP) pathogens in drug-dependent patients.

**Materials and methods:**

The susceptibility of community-acquired pneumonia pathogens isolated from sputum was studied in 31 drug-dependent patients. 13 (42%) were women and 18 (58%) were men. The average age of this group was 28.6 ± 1.2 years. The antimicrobial activity of the main groups of antibiotics was studied in comparison with the susceptibility of the archive *Staphylococcus aureus* ATCC25923 strain.

**Results:**

In total, 51 strains of microorganisms were isolated, among which *S. aureus* (19 strains, 37.3%) and fungi of the genus *Candida* (16 strains, 31.3%) prevailed, while in 15 cases, bacteria were inoculated in the form of monoculture, and in 16 cases in the form of associations of bacteria and fungi of the genus *Candida*. When studying the susceptibility of the isolated strains of microorganisms to the main groups of antibiotics, their high (*P* ≤ 0.05) resistance to the main groups of antimicrobial drugs used in standard treatment regimens for CAP was established.

**Conclusions:**

CAP in drug-dependent patients has a severe complicated course due to the combination of highly virulent bacterial and fungal infections. The causative agents of community-acquired pneumonia in drug-dependent patients have low susceptibility to the main groups of antibiotics used in the treatment of this pathology.

